# Refractive Index of Heavily Germanium-Doped Gallium Nitride Measured by Spectral Reflectometry and Ellipsometry

**DOI:** 10.3390/ma14237364

**Published:** 2021-11-30

**Authors:** Dario Schiavon, Robert Mroczyński, Anna Kafar, Grzegorz Kamler, Iryna Levchenko, Stephen Najda, Piotr Perlin

**Affiliations:** 1 Polish Academy of Sciences, Institute of High Pressure Physics, Al. Sokołowska 29/37, 01-142 Warsaw, Poland; ak@unipress.waw.pl (A.K.); grzegorz.kamler@unipress.waw.pl (G.K.); levchenko@unipress.waw.pl (I.L.); piotr@unipress.waw.pl (P.P.); 2 TOP-GAN sp.z o.o., Ul. Solec 24/90, 00-403 Warsaw, Poland; s.najda@topganlasers.com; 3 Institute of Microelectronics and Optoelectronics, Warsaw University of Technology, Ul. Koszykowa 75, 00-662 Warsaw, Poland; robert.mroczynski@pw.edu.pl

**Keywords:** gallium nitride, germanium doping, index of refraction, electron plasma, reflectometry, ellipsometry

## Abstract

Gallium nitride (GaN) doped with germanium at a level of 10^20^ cm^−3^ is proposed as a viable material for cladding layers in blue- and green-emitting laser diodes. Spectral reflectometry and ellipsometry are used to provide evidence of a reduced index of refraction in such layers. The refractive-index contrast to undoped GaN is about 0.990, which is comparable to undoped aluminium gallium nitride (AlGaN) with an aluminium composition of 6%. Germanium-doped GaN layers are lattice-matched to native GaN substrates; therefore, they introduce no strain, cracks, and wafer bowing. Their use, in place of strained AlGaN layers, will enable significant improvements to the production process yield.

## 1. Introduction

Semiconductor laser diodes find countless applications in telecommunication, medical/surgical systems, and other fields as compact high-power light sources that can be modulated quickly and are easily coupled to optical fibers. To ensure low-threshold currents and high efficiency of the laser, it is important to optimize the transversal confinement of the optical modes. In the case of horizontal cavity, edge-emitting laser diodes, the typical epitaxial structure includes layers of varying refractive indexes: a middle active layer with a given index of refraction, which is placed between two cladding layers with a lower index. In a waveguide, the optical modes tend to be stronger where the refractive index is higher, so the vertical profile of the refractive index confines the optical mode in the middle layer, which is where light amplification takes place.

Of all the semiconductor materials used for laser diodes, nitride semiconductors (AlGaInN) have established themselves as the most successful for efficient emission in the green–blue–ultraviolet range of the electromagnetic spectrum [1]. In this material system, increasing the amount of aluminium in the alloy is the preferred way to decrease the refractive index. Therefore, where the active layer must be made of the nitride alloy with the bandgap energy required to obtain the desired emission wavelength, the cladding layers require a considerably larger amount of aluminium to provide an adequate refractive-index contrast for mode confinement. For blue and green laser diodes, the typical amount of aluminium may range between 5% and 10%, which grants a refractive index contrast of about 0.992–0.984 (at 450 nm), with respect to GaN [2].

However, the stacking of layers of different alloys is made difficult by the lattice mismatch between the basic components AlN, GaN, and InN, which is particularly large in the nitrides, compared to other material systems. As a consequence of the biaxial strain, introduced by heteroepitaxial growth, layers that are thicker than a certain critical thickness relax with the formation of cracks and dislocations. So, one cannot just recreate the structure that is typical for arsenides (AlGaAs/GaAs/AlGaAs) and replace it with its nitride analogue (AlGaN/GaN/AlGaN) because, for Al compositions above 8% and with sufficient thickness (in the order of 1 μm), an AlGaN layer does not withstand the consequent stress [3]. Devices that are intersected by a crack are irremediably damaged, so the yield of the production process can be reduced considerably.

Even structures whose layers do not exceed their critical thickness can turn out to be unworkable because of the wafer bow induced by layer strain. The production process for laser diodes includes the use of photolithography, which is very sensitive to the curvature of the wafer. Light diffraction occurs in the areas where the photolithographic mask loses contact with the resist layer, making it difficult to produce uniform patterns across the bowed wafer. This limits the size of the processable wafers and further increases the production costs. Therefore, a means of controlling the refractive index (that does not require the use of aluminium) is highly desirable.

A different approach to reduce the refractive index of semiconductor layers is via the incorporation of a high concentration of dopant impurities, so as to produce a free-electron plasma. This approach is justified by the following three physical mechanisms. Firstly, in degenerately doped semiconductors, the Burstein–Moss effect (band filling) pushes the light-absorption edge to higher energies. Consequently, the refractive index is also shifted, due to the Kramers–Kronig relations, which connect the real and imaginary parts of complex functions that are analytic in the upper half-plane:

(1) Ren(ω)=1πP∫−∞+∞Imn(ω′)ω′−ωdω′.

The second effect is bandgap renormalization [4], which shifts the absorption edge to lower energies. It was shown to be negligible, compared to the Burstein–Moss effect, at doping levels above $9\;\times \;10^{18}$ ${\mathrm{cm}}^{-3}$ [5] and, therefore, will not be further considered in our study. The third effect is the decrease of permittivity caused by the coupling between the electromagnetic field and longitudinal oscillations of the electron plasma. In this region, neglecting the electron damping term, the background permittivity ε is changed into [6]:

(2) $$\begin{array}{ccc} \varepsilon^{\prime }\left(\omega \right)=\varepsilon \left(\omega \right)\left[1-{\left(\frac{\omega_{p}}{\omega }\right)}^{2}\right], & \; & \omega_{p}=\frac{N_{e}e^{2}}{m^{🟉}\varepsilon_{\infty }}, \end{array}$$

where $\omega_{p}$ is the resonance frequency of the electron plasma, $N_{e}$ is the electron density in the plasma, *e* the elementary charge, $m^{🟉}$ the electron effective mass, and $\epsilon_{\infty }$ the high-frequency permittivity limit in the medium. Note that, in wide-bandgap semiconductors, such as GaN, $\hbar \omega_{p}$ turns out to be much smaller than the bandgap energy, even at extremely high doping levels.

A possible disadvantage of employing heavy doping is the increase of light absorption via the excitation of the free electrons of the plasma, which could increase the optical losses in the laser resonator. However, free-carrier absorption is expected to be highest for photons with energy close to $\hbar \omega_{p}$ and should not be particularly strong for photons in the visible range. Besides, the corresponding optical losses would also be proportional to the overlap of the optical mode with the cladding layer, which can be minimized by proper optimization of the epitaxial structure. With confidence that free-carrier absorption can be managed, we leave an exact assessment for future research and focus, hereby, on the effect of heavy doping on the refractive index.

### Background Work

A first step in testing the use of heavy doping to lower the refractive index of GaN layers was accomplished in our group by doping GaN layers with oxygen at a level of 2 to $8\;\times \;10^{19}$ ${\mathrm{cm}}^{-3}$ [7,8,9]. The layers were grown with the high nitrogen pressure solution (HNPS) growth method [10] on a GaN substrate. Such a layer was then used as part of the lower cladding of a blue laser diode that showed no mode leakage into the substrate, despite the use of just a modest amount of aluminium in the remaining part of the lower cladding. This experiment demonstrated the potential of the doping technique. However, the heavily doped layer was grown in a different reactor than the rest of the laser structure, complicating the production process.

It would be preferable to grow the heavily doped layer by metal-organic vapor-phase epitaxy (MOVPE). Unfortunately, it was shown that the typical donor dopant used with MOVPE for GaN, which is silicon, cannot be incorporated in concentrations above $2\;\times \;10^{19}$ ${\mathrm{cm}}^{-3}$ without deteriorating the growth surface to an extent that no successive layer could be grown [11]. A solution to this problem came with germanium, an alternative dopant that offers an activation energy [12] and carrier mobility [13,14], similar to silicon, without compromising the surface morphology [11,14] or introducing strain [11,15], up to a concentration of at least $3\;\times \;10^{20}$ ${\mathrm{cm}}^{-3}$.

**Figure 1:**
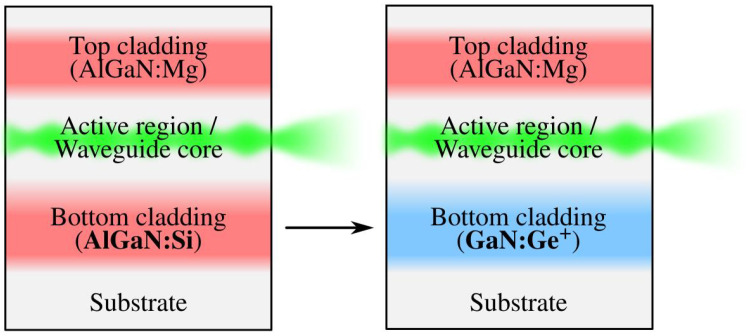
We aim at replacing the lower silicon-doped AlGaN cladding layer of the classic laser-diode structure with a heavily germanium-doped GaN layer.

Recently, we have been growing thick high-quality germanium-doped GaN layers by MOVPE [14]. In this article, we verify that a layer doped at the level of $10^{20}$ ${\mathrm{cm}}^{-3}$ indeed has a lower refractive index than undoped (or lightly doped) GaN and can, therefore, replace AlGaN as the material of the cladding layers in blue and green nitride laser diodes (Figure 1). Reflectometry and ellipsometry measurements both confirm our expectations, opening the way to new future experimentation.

## 2. Materials and Methods

### 2.1. Epitaxial Growth

The examined sample (Sample A) was grown on a GaN substrate using an Aixtron MOVPE reactor of the close coupled showerhead (CCS) design. The substrate itself, which has a thickness of approximately 300 μm and an unpolished back side, was grown by hydride vapor-phase epitaxy (HVPE) and doped with oxygen at a level of $2\;\times \;10^{18}$ ${\mathrm{cm}}^{-3}$. The MOVPE-grown structure consists of two layers: a 1-μm GaN layer, which is silicon-doped at a level of $4\;\times \;10^{18}$ ${\mathrm{cm}}^{-3}$, and a 1-μm GaN layer, which is germanium-doped at a level of approximately $10^{20}$ ${\mathrm{cm}}^{-3}$. Only the latter layer has a doping concentration high enough to produce an appreciable change in the refractive index, so the other layers can be considered equivalent to completely undoped GaN, as far as the refractive index is concerned.

The Ge-doped layer was grown at a temperature of 970 °C (as measured at the susceptor surface by an Aixtron Argus™ pyrometer) and a pressure of 10 kPa, with a gap distance of 6 mm between the susceptor and showerhead. The total flow into the reactor was 8 slm, of which 2 slm of ammonia (NH${}_{3}$), 25 μmol/min of trimethylgallium [Ga(CH_3_)_3_], and 7.6 μmol/min of germane (GeH${}_{4}$) were precursors for nitrogen, gallium, and germanium atoms, respectively. The remaining flow was H${}_{2}$. The growth rate was 0.80 μm/h, as determined by in-situ laser reflectometry.

### 2.2. Preliminary Characterization

**Figure 2:**
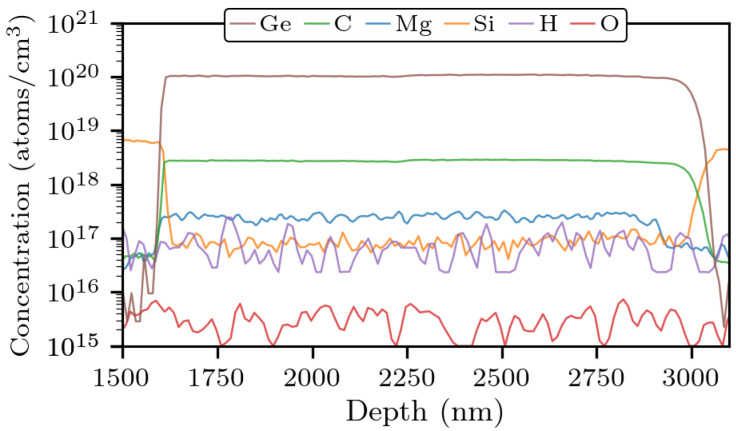
Impurity concentrations in Sample B, measured by secondary-ion mass spectrometry (SIMS) at EAG Laboratories. The germanium-doped GaN layer is located at depths in the range of 1600–3000 nm. The layers above and below are not of interest.

The germanium concentration of the layer is assessed on the base of secondary-ion mass spectrometry (SIMS) measurements, performed on a separate sample (Sample B), where a germanium-doped layer was grown in the same conditions as above, except for the temperature, which was 5 °C higher. In Sample B, the concentration was determined to be between 1.0 and $1.1\;\times \;10^{20}$ ${\mathrm{cm}}^{-3}$ (Figure 2). As germanium incorporation is quite sensitive to temperature and more efficient at lower temperature [14], we take $10^{20}$ ${\mathrm{cm}}^{-3}$ as a lower bound for the germanium concentration in Sample A. The SIMS measurement also evidenced that the layer contains carbon impurities at a concentration of $2.8\;\times \;10^{20}$ ${\mathrm{cm}}^{-3}$, which is expected, given the growth conditions employed, especially the low pressure [16] and low ammonia flow [17]. Anyway, since the carbon concentration is almost two orders of magnitude lower than germanium, no significant doping compensation should occur.

**Figure 3:**
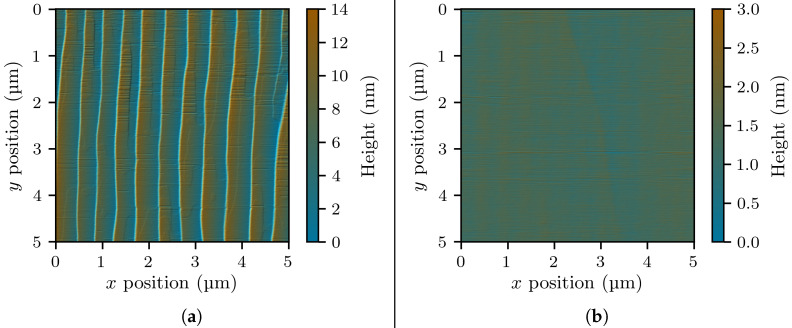
Atomic-force microscopy (AFM) scans of our germanium-doped GaN layer immediately before (**a**) and after (**b**) chemical mechanical polishing (CMP).

The surface of Sample A was examined by atomic-force microscopy (AFM). The resulting scan is shown in Figure 3a. The surface was mostly free of V-pits [18,19]; however, a high degree of step bunching [20,21] led to the formation of macrosteps of an average height of 13 nm, leading to a roughness $R_{q}$ of 2.67 nm. Convinced that the macrosteps would needlessly complicate our reflectometry and ellipsometry measurements, we opted to remove about 15 nm of the layer with a silica-based chemical mechanical polishing (CMP) process, optimized to produce smooth surfaces ready for further epitaxial growth (similar to [22]). After cleaning in hydrofluoric acid and piranha solution, AFM scans of the resulting surface do not show any steps (Figure 3b), and the roughness $R_{q}$ is reduced to 0.13 nm.

### 2.3. Reflectometry

Sample A was analyzed by spectral reflectometry, using a home-made system based on a fiber-optic reflection probe bundle, in the wavelength range from 200 to 900 nm. A pristine GaN substrate (Sample R) was also measured as a reference sample. We proceeded by fitting the ratio with a theoretical model, calculated from the complex refractive index and thickness of each layer of the structure. The reflection and transmission coefficients $r_{i}$ and $t_{i}$, of the *i*-th interface, are given by the Fresnel equations. The overall reflection and transmission *r* and *t*, for the whole structure, are then calculated using the transfer-matrix method:

(3) $$\left(\begin{array}{c} 1\\ r \end{array}\right)=\left[\prod_{i}\;\frac{1}{t_{i}}\left(\begin{array}{cc} 1 & r_{i}\\ r_{i} & 1 \end{array}\right)\left(\begin{array}{cc} e^{-jk_{i}d_{i}} & 1\\ 1 & e^{+jk_{i}d_{i}} \end{array}\right)\right]\left(\begin{array}{c} t\\ 0 \end{array}\right),$$

where $k_{i}=\frac{n_{i}E}{\hbar c}$ and $d_{i}$ are the complex wavevector (in the direction normal to the layers) and the thickness of the *i*-th layer.

**Figure 4:**
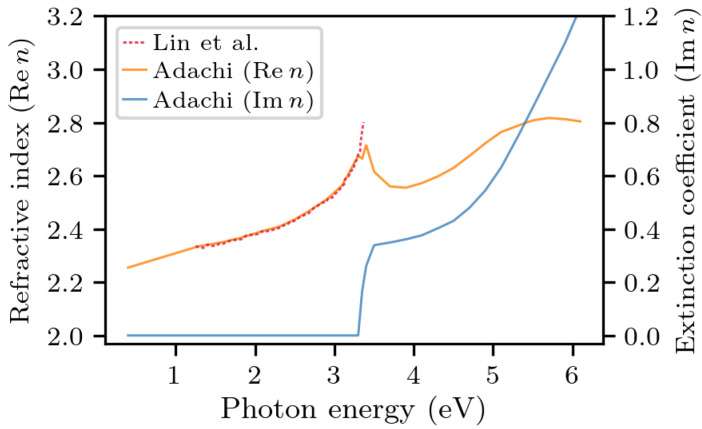
The real and imaginary parts of the refractive index of undoped GaN. The data are taken from Lin et al. [23] and Adachi [24]. In our analysis, we will be using Adachi’s data.

To apply this model, the refractive index of each layer must be made explicit. Since the GaN substrate is not heavily doped, its refractive index n(E) (a function of energy) is assumed to be equal in to what is reportedthe literature for undoped GaN [23,24]; the real and imaginary parts of the refractive index are shown in Figure 4, as a function of photon energy. As we see, two different sources show good agreement, which builds confidence about their accuracy. In this work, we used the data from Adachi [24], since they were measured across a larger range of energies.

The refractive index of the germanium-doped layer is derived from that of undoped GaN, by including two effects produced by the high doping level: the Burstein–Moss and plasmonic effects. For simplicity, we assume that the Burstein–Moss effect induces a rigid translation of the refractive index towards higher energies:

(4) $$n^{\prime }\left(E\right)=n\left(E-{\Delta E}_{bm}\right),$$

where ${\Delta E}_{bm}$ is the Burstein–Moss shift. Note that a rigid translation preserves the Kramer–Kronig relations, as long as it applies to both the real and imaginary parts. This may not be the most theoretically rigorous method to account for the Burstein–Moss effect, but it should work reasonably well for energies below the bandgap. As for the plasmonic effect, we assume it, too, changes the refractive index, according to the formula:

(5) $$n^{\prime \prime }\left(E\right)=n^{\prime }\left(E\right)\sqrt{1-{\left(\frac{\hbar \omega_{p}}{E}\right)}^{2}},$$

which follows from Equation (2). The parameters ${\Delta E}_{bm}$ and $\hbar \omega_{p}$, therefore, become the fitting parameters of our model and are optimized with the least squares method (so as to minimize the squared error to the reflectometry data). The optimization was applied only for wavelengths above 350 nm because we were not able to find a good fit for energies above the bandgap, probably due to the limitations of our model. The results are reported in the next section.

### 2.4. Ellipsometry

Sample A was also analyzed by a Horiba Jobin Yvon UVISEL 2™ ellipsometer, in the range of visible wavelengths from 300 to 850 nm, with 1-nm steps and at a nominal angle of $\vartheta =70^{{}^{\circ}}$ from the normal to the surface of the sample. The sample is illuminated with a beam of white light, whose *s*- and *p*-components are equal and in-phase at each wavelength (the two components being defined, respectively, as perpendicular and parallel to the incidence plane). The reflected beam is spectrally analyzed. The ellipsometric parameters Ψ and Δ codify the amplitude ratio and the phase shift of the corresponding components $r_{s}$ and $r_{p}$ of the reflected light:

(6) $$\begin{array}{ccc} \Psi =\arctan\left|\frac{r_{p}}{r_{s}}\right| & \; & \Delta =\arg\left(\frac{r_{p}}{r_{s}}\right) \end{array}$$

The Ψ and Δ curves are then fitted with the same model that was described for reflectometry, except it was adapted to distinguish between *s*- and *p*-polarization and to account for the angled illumination (so the Fresnel coefficients change and we have $k_{i}=\;\sqrt{n_{i}^{2}\;-\;\sin^{2}\;\vartheta }\;\frac{E}{\hbar c}$ in Equation (3)). Once again, least squares optimization was applied only for wavelengths above 350 nm, and the parameters that have been optimized by the fitting procedure are the Burstein–Moss energy shift ${\Delta E}_{bm}$ and plasma frequency $\hbar \omega_{p}$.

## 3. Results

**Figure 5:**
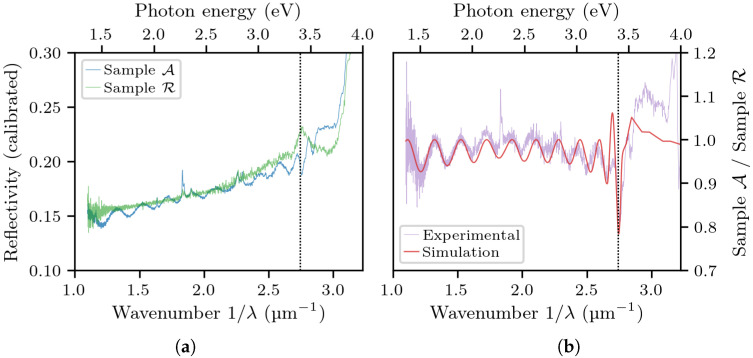
The reflectivity spectra of sample A and R (**a**) and their ratio with the simulation superimposed (**b**). The vertical line marks the bandgap energy of undoped GaN.

**Figure 6:**
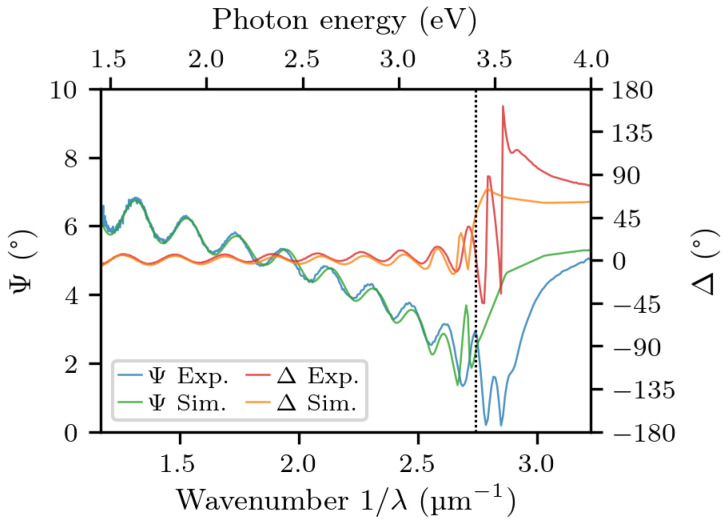
The ellipsometric parameters Ψ and Δ of sample A, as a function of wavenumber, with the simulation superimposed. The vertical line marks the bandgap energy of undoped GaN.

The reflection spectra from reflectometry (with the background light subtracted from them) are shown in Figure 5a. Their ratio, adequately fitted with our model, is reported in Figure 5b. The resulting fitting parameters were ${\Delta E}_{bm}=0.128$ eV and $\hbar \omega_{p}=0.246$ eV. Note that, even if the simulated curve does not perfectly match the height of the experimental curve, it does match the amplitude of the oscillations, which is what matters the most for the assessment of the refractive index contrast. The corresponding results from ellipsometry are shown in Figure 6. In this case, the optimal fitting parameters were ${\Delta E}_{bm}=0.059$ eV and $\hbar \omega_{p}=0.278$ eV.

For comparison, previous measurements of the refractive index of a heavily Ge-doped GaN layer performed by others [25] by infrared ellipsometry determined a plasma frequency of $\hbar \omega_{p}=0.434$ eV for a doping level of $2.4\;\times \;10^{20}$ ${\mathrm{cm}}^{-3}$, which would correspond to $\hbar \omega_{p}=0.181$ eV for the doping level of Sample A ($10^{20}$ ${\mathrm{cm}}^{-3}$).

In both cases, the thickness of the germanium-doped layer was fixed at 985, resulting in the correct fit of the period of the Fabry–Pérot oscillations (i.e., the distance between the peaks). We know that the value is correct because it corresponds to the expected thickness of the grown germanium-doped layer (1 μm), minus the thickness that was lost to CMP (∼15 nm). In the case of ellipsometry, the optimal value of the incidence angle ϑ was determined to be 70.3°, very close to the nominal 70°.

## 4. Discussion

**Figure 7:**
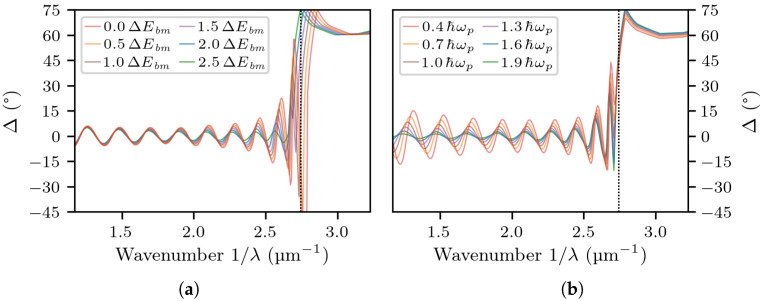
The simulated ellipsometric parameter Δ for the structure of Sample A, obtained by decreasing or increasing the parameters ${\Delta E}_{bm}$ (**a**) and $\hbar \omega_{p}$ (**b**). Note that the former affects the Δ curve most at high energies (i.e., close to the bandgap), whereas the latter affects the Δ curve most at low energies (i.e., closer to the plasma frequency $\hbar \omega_{p}$).

As we have determined, by adjusting the fitting parameters ${\Delta E}_{bm}$ and $\hbar \omega_{p}$ manually, both have a clear effect on the amplitude of the Fabry–Pérot oscillations for both reflectometry and ellipsometry (both Ψ and Δ). An increase of ${\Delta E}_{bm}$ results in wider oscillations at energies close to the bandgap (Figure 7a), whereas an increase of $\hbar \omega_{p}$ results in wider oscillations at energies farther away from the bandgap (Figure 7b). Neither of the two parameters change the period or the vertical position of the oscillations very much, as those are primarily affected by the thickness of the heavily-doped layer and incidence angle ϑ, respectively. In effect, the fitting procedure computes the values that best match the amplitude of the oscillations in the whole energy range up to the bandgap. In all cases, we deem the fits satisfactory.

**Figure 8:**
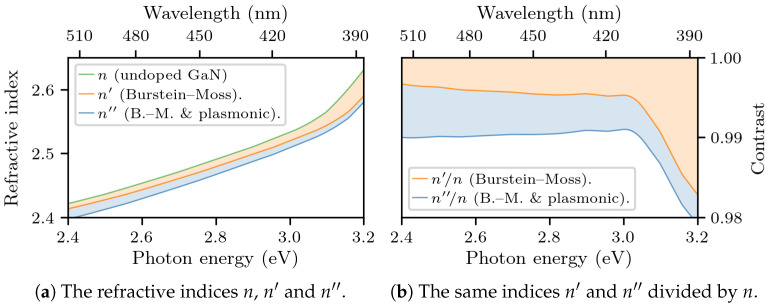
The refractive index *n* of undoped GaN (from [24]), as well as the decrease caused by the Burstein–Moss (orange area) and plasmonic effects (blue area), leading to the modified indices $n^{\prime }$ and $n^{\prime \prime }$ (calculated from our model using the fitting parameters from ellipsometry: ${\Delta E}_{bm}=0.059$ eV, $\hbar \omega_{p}=0.278$ eV).

The theoretical model, together with the parameters obtained via the fitting procedure described above, allows us to estimate the refractive index as a function of photon energy, as well as the relative contributions of the Burstein–Moss and plasmonic effects on the overall doping-induced decrease of the refractive index. Figure 8 shows the results, in the case of the ellipsometry data. Coincidentally, in the range of 2.75–3 eV (corresponding to the wavelengths from 450 to 410 nm), the two effects contribute equally: $\frac{n^{\prime }}{n}=\frac{2.470}{2.481}=0.995$ and $\frac{n^{\prime \prime }}{n^{\prime }}=\frac{2.457}{2.470}=0.995$.

**Figure 9:**
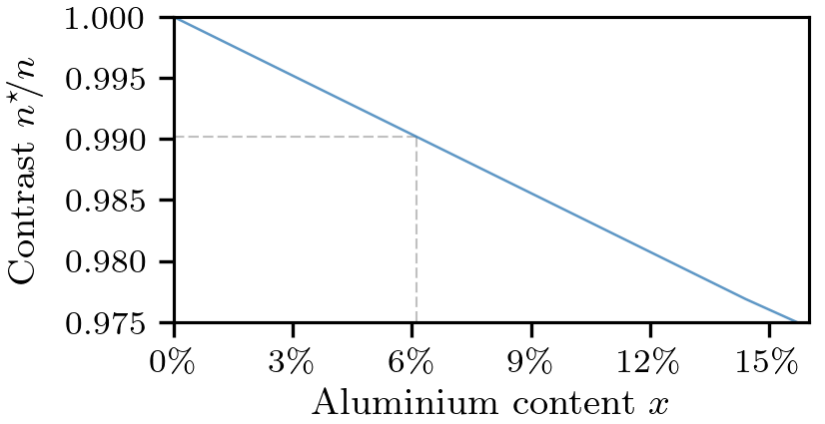
The refractive index contrast of Al${}_{x}$Ga${}_{1-x}$N, with respect to GaN as a function of the aluminium content *x* (data extrapolated from [2]). A contrast of 0.990 corresponds to an aluminium content of 6%.

Specifically, at the wavelength of 450 nm, which we consider of most practical interest, the refractive-index contrast between heavily doped and undoped GaN is determined to be $\frac{n^{\prime \prime }}{n}=\frac{2.448}{2.481}=0.987$ for reflectometry and $\frac{n^{\prime \prime }}{n}=\frac{2.457}{2.481}=0.990$ for ellipsometry. While the resulting values between the measurement techniques are not overly different, we consider ellipsometry to be more accurate. According to similar ellipsometry-based measurements previously performed by others [2], a contrast of 0.990 corresponds to that between Al${}_{x}$Ga${}_{1-x}$N and GaN, where the aluminium content *x* is 6% (Figure 9), meaning that layers doped with germanium, at a level of $10^{20}$ ${\mathrm{cm}}^{-3}$, can seamlessly replace undoped (or moderately-doped) Al${}_{0.06}$Ga${}_{0.94}$N layers in nitride-based laser structures, with no change of the refractive index profile.

## 5. Conclusions

A GaN layer doped with germanium at a level of $10^{20}$ ${\mathrm{cm}}^{-3}$ was grown by MOVPE on a moderately doped GaN substrate. The layer was analyzed by both reflectometry and ellipsometry techniques to measure the refractive-index contrast at the interface between the germanium-doped layer and substrate. The contrast was determined to be 0.990 or lower. To obtain the same contrast with an AlGaN layer, an aluminium content of 6% would have been necessary. The approach of doping with germanium has the undoubted advantage of not introducing a biaxial strain in the structure (and, thus, no cracks or wafer bow). For this reason, we consider germanium-doped GaN a better material for the cladding layers in the production of blue and green nitride laser diodes.

## Data Availability

Data is contained within the article.

## References

[1] Schwarz U.T. (2021). Physics and Technology of AlGaInN-Based Laser Diode. Digital Encyclopedia of Applied Physics.

[2] Sanford N.A., Robins L.H., Davydov A.V., Shapiro A., Tsvetkov D.V., Dmitriev A.V., Keller S., Mishra U.K., DenBaars S.P. (2003). Refractive index study of Al_x_Ga_1-x_N films grown on sapphire substrates. J. Appl. Phys..

[3] Sarzyński M., Kryśko M., Targowski G., Czernecki R., Sarzyńska A., Libura A., Krupczyński W., Perlin P., Leszczyński M. (2006). Elimination of AlGaN epilayer cracking by spatially patterned AlN mask. Appl. Phys. Lett..

[4] Berggren K.F., Sernelius B.E. (1981). Band-gap narrowing in heavily doped many-valley semiconductors. Phys. Rev. B.

[5] Feneberg M., Osterburg S., Lange K., Lidig C., Garke B., Goldhahn R., Richter E., Netzel C., Neumann M.D., Esser N. (2014). Band gap renormalization and Burstein-Moss effect in silicon- and germanium-doped wurtzite GaN up to 10^20^*cm*^−3^. Phys. Rev. B.

[6] Peter Y.U., Cardona M. (2010). Fundamentals of Semiconductors: Physics and Materials Properties.

[7] Perlin P., Holc K., Sarzyński M., Scheibenzuber W., Marona L., Czernecki R., Leszczyński M., Bockowski M., Grzegory I., Porowski S. (2009). Application of a composite plasmonic substrate for the suppression of an electromagnetic mode leakage in InGaN laser diodes. Appl. Phys. Lett..

[8] Perlin P., Czyszanowski T., Marona L., Grzanka S., Kafar A., Stanczyk S., Suski T., Leszczyński M., Boćkowski M., Muzioł G., Chyi J.I., Nanishi Y., Morkoç H., Piprek J., Yoon E. (2012). Highly Doped GaN: A Material for Plasmonic Claddings for Blue/Green InGaN Laser Diodes.

[9] Stańczyk S., Czyszanowski T., Kafar A., Czernecki R., Targowski G., Leszczyński M., Suski T., Kucharski R., Perlin P. (2013). InGaN laser diodes with reduced AlGaN cladding thickness fabricated on GaN plasmonic substrate. Appl. Phys. Lett..

[10] Bockowski M. (2014). High nitrogen pressure solution growth of GaN. Jpn. J. Appl. Phys..

[11] Fritze S., Dadgar A., Witte H., Bügler M., Rohrbeck A., Bläsing J., Hoffmann A., Krost A. (2012). High Si and Ge n-type doping of GaN doping–Limits and impact on stress. Appl. Phys. Lett..

[12] Götz W., Kern R.S., Chen C.H., Liu H., Steigerwald D.A., Fletcher R.M. (1999). Hall-effect characterization of III–V nitride semiconductors for high efficiency light emitting diodes. Mater. Sci. Eng. B.

[13] Oshima Y., Yoshida T., Watanabe K., Mishima T. (2010). Properties of Ge-doped, high-quality bulk GaN crystals fabricated by hydride vapor phase epitaxy. J. Cryst. Growth.

[14] Schiavon D., Litwin-Staszewska E., Jakieła R., Grzanka S., Perlin P. (2021). Effects of MOVPE Growth Conditions on GaN Layers Doped with Germanium. Materials.

[15] Nenstiel C., Bügler M., Callsen G., Nippert F., Kure T., Fritze S., Dadgar A., Witte H., Bläsing J., Krost A. (2015). Germanium—The superior dopant in n-type GaN. Phys. Status Solidi RRL.

[16] Gamarra P., Lacam C., Tordjman M., Splettstösser J., Schauwecker B., di Forte-Poisson M.A. (2015). Optimisation of a carbon doped buffer layer for AlGaN/GaN HEMT devices. J. Cryst. Growth.

[17] Ciarkowski T., Allen N., Carlson E., McCarthy R., Youtsey C., Wang J., Fay P., Xie J., Guido L. (2019). Connection between Carbon Incorporation and Growth Rate for GaN Epitaxial Layers Prepared by OMVPE. Materials.

[18] Chen Y., Takeuchi T., Amano H., Akasaki I., Yamada N., Kaneko Y., Wang S.Y. (1998). Pit formation in GaInN quantum wells. Appl. Phys. Lett..

[19] Knetzger M., Meissner E., Schröter C., Friedrich J. (2019). Theoretical aspects and microstructural investigations on V-pit defects in HVPE grown GaN. J. Cryst. Growth.

[20] Hiramatsu K., Amano H., Akasaki I., Kato H., Koide N., Manabe K. (1991). MOVPE growth of GaN on a misoriented sapphire substrate. J. Cryst. Growth.

[21] Ramana Murty M.V., Fini P., Stephenson G.B., Thompson C., Eastman J.A., Munkholm A., Auciello O., Jothilingam R., DenBaars S.P., Speck J.S. (2000). Step bunching on the vicinal GaN(0001) surface. Phys. Rev. B.

[22] Hanser D., Tutor M., Preble E., Williams M., Xu X., Tsvetkov D., Liu L. (2007). Surface preparation of substrates from bulk GaN crystals. J. Cryst. Growth.

[23] Lin M.E., Sverdlov B.N., Strite S., Morkoç H., Drakin A.E. (1993). Refractive Indices of Wurtzite and Zincblende GaN. Electron. Lett..

[24] Adachi S. (2013). Optical Constants of Crystalline and Amorphous Semiconductors: Numerical Data and Graphical Information.

[25] Kirste R., Hoffmann M.P., Sachet E., Bobea M., Bryan Z., Bryan I., Nenstiel C., Hoffmann A., Maria J.P., Collazo R. (2013). Ge doped GaN with controllable high carrier concentration for plasmonic applications. Appl. Phys. Lett..

